# Quetiapine Modulates Histone Methylation Status in Oligodendroglia and Rescues Adolescent Behavioral Alterations of Socially Isolated Mice

**DOI:** 10.3389/fpsyt.2019.00984

**Published:** 2020-01-31

**Authors:** Xianjun Chen, Hao Liu, Jingli Gan, Xiaorui Wang, Guangdan Yu, Tao Li, Xuejun Liang, Bin Yu, Lan Xiao

**Affiliations:** ^1^ Department of Histology and Embryology, Institute of Brain and Intelligence, Army Medical University (Third Military Medical University), Chongqing, China; ^2^ Department of Psychiatry, Mental Diseases Prevention and Treatment Institute of PLA, No.988 Hospital of Joint Logistic Support Force of PLA, Jiaozuo, China; ^3^ Department of Neurosurgery, Xinqiao Hospital, Third Military Medical University, Chongqing, China

**Keywords:** quetiapine, social isolation, myelin, oligodendroglia, histone methylation, mental illness

## Abstract

Epigenetic alterations and impaired oligodendroglial myelination in the prefrontal cortex have been shown to correlate with behavioral and cognitive dysfunctions in social deprivation. Our previous study demonstrated that quetiapine, an atypical antipsychotic, could promote oligodendroglial differentiation and myelination. However, whether and how quetiapine could be beneficial in modulating aberrant epigenetic alterations in oligodendroglial cells and relieving behavioral alterations from social isolation is unknown. In this study, quetiapine was orally administered in adolescent mice undergoing mild stress of social isolation. We firstly confirmed that social isolation during a novel adolescent period could impair sociability, but not locomotive behaviors in mice. Moreover, quetiapine alleviated myelin deficits, and increased levels of histone methylation (H3K9me3) in mature oligodendroglia in the prefrontal cortex of socially isolated mice. Strikingly, quetiapine treatment significantly increased locomotive activity, and successfully reversed social avoidance behavior of the socially isolated mice. Taken together, our data suggest that quetiapine may rescue behavioral changes from social isolation through modulating epigenetic status toward the beneficial direction for oligodendroglial maturation, providing new insights into the pharmacological mechanism of quetiapine for mental illnesses.

## Introduction

Social deprivation, that could induce mild cognitive impairment, impulsivity and social deficits (1, 2), has been well-recognized as an environmental risk factor of psychiatric disorders (3). Some clinical findings showed that social deprivation of children was correlated with aberrant alterations in white matter tracts (4, 5). Furthermore, some rodent studies demonstrated that social isolation during the critical period of adolescence as well as adult age resulted in aberrant changes of oligodendroglial (OL) gene expression, OL morphology, and myelin thickness in the prefrontal cortex (PFC) (6, 7). Even though OL development and myelination are highly dynamic processes known to be regulated by experience and neuronal activity (8, 9), however, the underlying mechanism of how social isolation lead to OL and myelin deficits is still unknown.

Recently, multiple environmental risk factors have been identified to induce abnormal epigenetic alterations in the brains of schizophrenia cohorts (10), including DNA methylation, histone modification, and dysregulation of miRNAs (11). To be noted, the largest genome-wide association study of schizophrenia found that the dysregulated genes were highly enriched in the epigenetic control of OL lineage cells (12, 13). As we know, the transition from OL progenitor cells to mature OLs is characterized by a rapid and substantial chromatin remodeling, which is highly governed by epigenetic regulators including histone modification and DNA methylation factors (7, 14, 15). More specifically, some rodent studies demonstrated that social isolation could result in aberrant histone methylation changes in OLs (7). While clemastine reversed histone methylation in OLs, promote myelination in the PFC and rescued behavioral changes of social isolation (16), raising an intriguing possibility that epigenetic status of OLs could be a novel target for drug treatment.

Quetiapine, a Food and Drug Administration-approved atypical antipsychotic, has been shown to promote oligodendroglial progenitor cell (OPC) differentiation and remyelination (17–19). Furthermore, quetiapine treatment showed a beneficial effect in improving cognitive functions, such as the spatial working memory, in cuprizone-induced demyelinating mice model (18). However, whether quetiapine could be beneficial in modulating histone methylation status in OL, and recovering the myelin deficits as well as behavioral alterations from social isolation is unknown.

To address this issue, we employed a mouse model of social isolation and administered quetiapine in socially isolated mice during adolescence. We found that quetiapine could restore myelin deficits in PFC of socially isolated mice. Furthermore, quetiapine induced higher levels of repressive histone methylation (H3K9me3) in OLs, providing a possible mechanism that quetiapine could promote OLs differentiation through regulating chromatin compaction. Moreover, we confirmed that social isolation during adolescence (postnatal day 21 to P56) could impair sociability in mice. Strikingly, quetiapine treatment significantly enhanced locomotive activity, and successfully rescued the social avoidance behavior in socially isolated mice. Taken together, our data suggest a positive effect of quetiapine on reversing myelin deficits and rescuing behavioral abnormalities in socially isolated mice.

## Materials and Methods

### Social Isolation and Drug Treatment

All experimental C57BL/6J mice were maintained in a temperature and humidity controlled environment on a 12 h light/dark cycle with free access to food and water. The experimental mice included in this study were randomly selected without any preference of mice gender. The ratio of male to female mice was approximately 1:1. For social isolation experiment, mice were singly housed from P21 to P56. Otherwise, mice were group housed (3–4 animals per cage) in a regular environment. For the drug treatment, mice were orally given vehicle (double-distilled H2O, ddH2O) or quetiapine [dissolved in ddH2O, 10 mg/(kg.day)] for 3 weeks (P35 to P56) while continuing to be housed in the isolated environment.

### Immunostaining

Mice for each group were deeply anesthetized with 1% sodium pentobarbital and transcardially perfused with 4% paraformaldehyde in PBS. Brains were dehydrated in 10, 20, and 30% sucrose in 4% paraformaldehyde for 12 hours, respectively. The frozen brains were sectioned (20 μm) on acryostat microtome (MS1900, Leica). Immunostaining was performed as previously described (20). Free-floating sections were incubated with primary antibodies overnight at 4°C after 1 h blocking with 5% BSA, followed by secondary antibodies incubation (1.5 h). Primary antibodies included: mouse anti-APC (1:500, OP-80, Millipore), rabbit anti-H3K9me3 (1:500, ab8898, Abcam), and mouse anti-MBP (1:200; Santa Cruz). Alexa fluor 488-conjugated secondary antibodies against mouse and 568-conjugated secondary antibodies against rabbit (1:2000; Invitrogen) were used.

### RNA Extraction and Analysis

RNA extraction, reverse-transcription, and quantitative RT-PCR (RT-qPTR) were performed as previously described (21). Primers used are provided below. MBP: forward 5′-ACACACGAGAACTACCCATTATGG-3′, reverse 5′-AGAAATGGACTACTGGGTTTTCATCT-3′. MAG: forward 5′- GACTAAGCCCTAGCTCAATCAC-3′, reverse 5′- CCCTCGAGA AGCTGAAATCAT-3′. GAPDH: forward 5′- ACCCAGAAGACTGTGGATGG-3′, and reverse 5′- CACATTGGGGGTAGGAACAC-3′.

### Image Acquisition and Quantification

Fluorescent images from brain slices were collected on a confocal microscope (Olympus, IV 1000, Shinjuku, Tokyo) with the excitation wavelengths appropriate for AlexaFluor 488 (488 nm), 596 (568 nm), or DAPI (380 nm). For the quantification of MBP staining, a same layer of brain slices was scanned under the same condition. The MBP fluorescence positive area was analyzed by Image-Pro Plus software 5.0 (Media Cybernetics). Besides, the pixel intensity of H3K9me3 was quantified using Image-Pro Plus software 5.0 (Media Cybernetics). For statistical analysis, at least three representative images (20×) were randomly acquired from each mouse, and six mice were included in each treatment condition.

### Behavioral Tests

Open field test was performed in an open-field apparatus (Biowill, Shanghai, China) as previously described (22). Briefly, mice were placed in the center of the open-field box (50 cm × 50 cm × 50 cm). Then the mice activity was recorded with a digital video camera (digital CCD camera, Sony) during a period of 10 min. The total and center-area traveled distances were measured and the time spent in the central area was recorded.

Social interaction test was conducted as previously described with a slight modification (23). Two identical plastic square cylinders (each 8 cm diameter, 12 cm tall) were set in opposite corners of a box (50 cm × 50 cm × 50 cm). Each cylinder was perforated with multiple holes (0.5 cm diameter) to allow for air exchange between the interior and exterior of the cylinder. The subject mouse was first placed at the center of the box. After a 10 min adaptation period in which the subject was free to explore each cylinder, a stranger mouse was placed in one of the cylinders. Containing the stranger mouse in the cylinder ensured that all social approach was initiated by the subject mouse without direct physical contact. The total distance travelled and time spent by the subject mouse on approaching and exploring each cylinder was measured for 5 min. After each experiment, all the apparatuses were wiped clean with 70% ethanol to remove traces of the previous assay.

### Statistical Analysis

We performed statistical analyses by using the GraphPad Prism 5 software (GraphPad Software Inc.). For between-group comparisons, we used the Independent-samples t-test. The Welch correction was applied if the variance was unequal. We considered results to be significant at p < 0.05.

## Results

### Social Isolation During Adolescence Leads to Impairment of Sociability in Adult Mice

**Figure 1:**
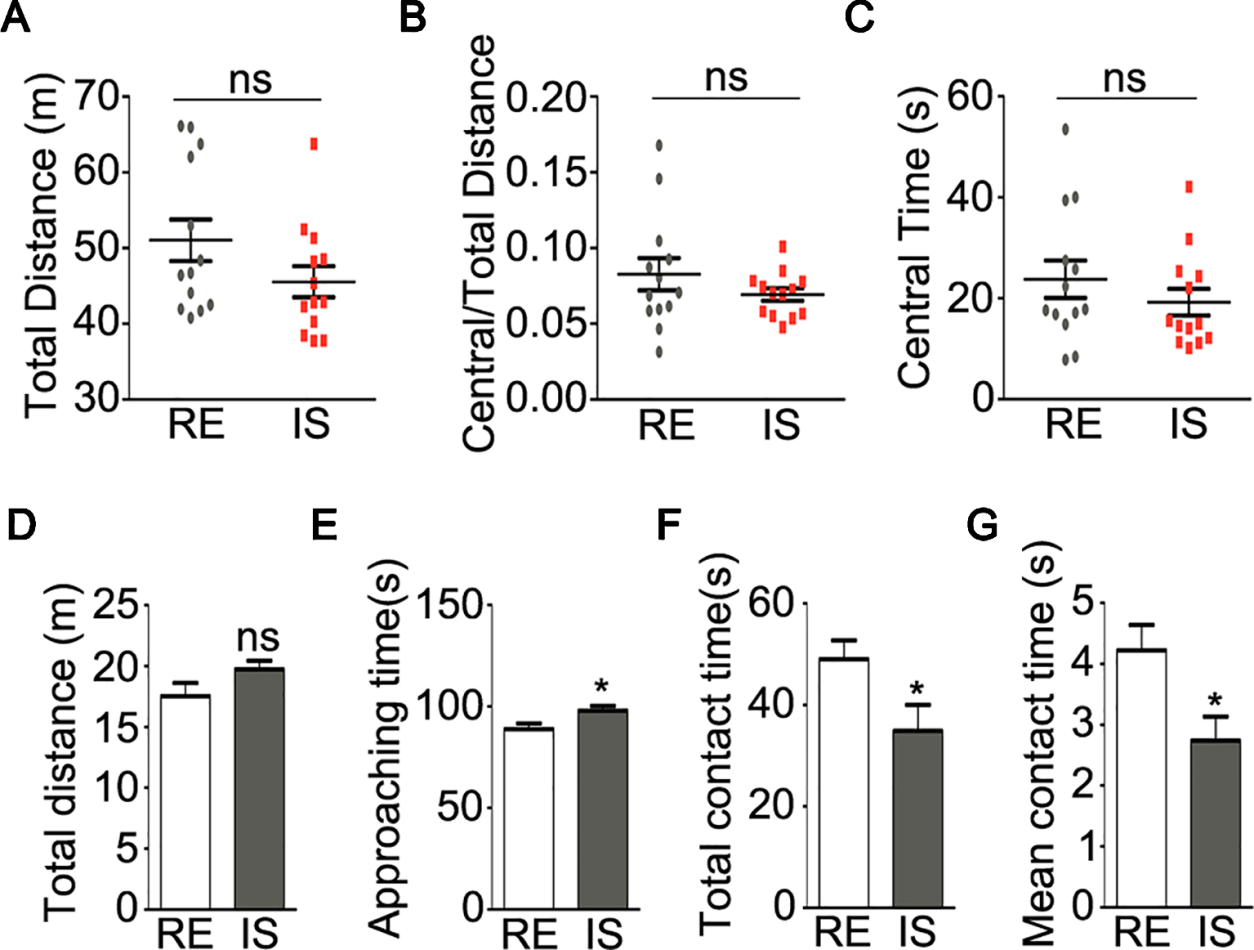
Behavioral analyses of socially isolated mice. **(A–C)** locomotor activity of mice in open field test (n = 13 for each group). Graphs show total distance **(A)**, ratio of central to total distance **(B)** and center time **(C)** in tested mice. **(D–G)** sociability of mice in social interaction test (n = 13 for RE group, n = 12 for IS group). Graphs show the total distance traveled **(D)**, duration of approaching to the stranger mice **(E)**, duration of total contact **(F)** and mean duration for each contact **(G)** in tested mice. RE, group-housed in regular environment; IS, social isolation. Data are expressed as mean ± SEM. *P < 0.05; ns, no significant difference.

To be noted, the peak onset of psychiatric disorders has been concentrated in a very narrow age range from adolescence to young adult (24). Some previous study found that impact of social isolation on mice behaviors occurred at a key period from P21 to P35 (6). In this study, mice after weaning (P21) were socially isolated for 5 weeks. Then the maladaptive behaviors after social isolation were tested at P56. As impaired locomotion has been considered as one of the key features of major psychiatric disorders, like schizophrenia (10), we thus performed the open field test, a non-conditioned procedure commonly used for assessing locomotive activity in rodents (25). In the open field test, socially isolated mice showed a trend of lower locomotive activity by traveling a shorter total distance during a 10-minute test as compared with group-housed mice in a regular environment, even though no significant difference was achieved (**Figure 1A**, P = 0.123). In addition, no significant difference was found in the ratio of travel distance within central area to total travel distance (**Figure 1B**, P = 0.257) or travel time in central area (**Figure 1C**, P = 0.329) between these two groups. These results suggest that social isolation during adolescence could not cause anxious phenotype, but may potentially impair locomotive activity at adult age.

Social interaction test was widely used to analyze sociability in mice (26). In the present study, as compared with group-housed mice, the socially isolated mice showed similar total traveled distance (**Figure 1D**, P = 0.106). The duration of socially isolated mice approaching the stranger mice was slightly but still significantly longer than the group housed mice (**Figure 1E**, P = 0.019), however, the isolated mice showed significantly shorter duration of contacting with the stranger mice (**Figure 1F**, P = 0.034) and shorter duration for each social contact (**Figure 1G**, P = 0.017), as compared with group-housed mice. These results strongly implied aberrant sociability in socially isolated mice.

### Quetiapine Reverses Myelin Deficit in the PFC of Socially Isolated Mice

**Figure 2:**
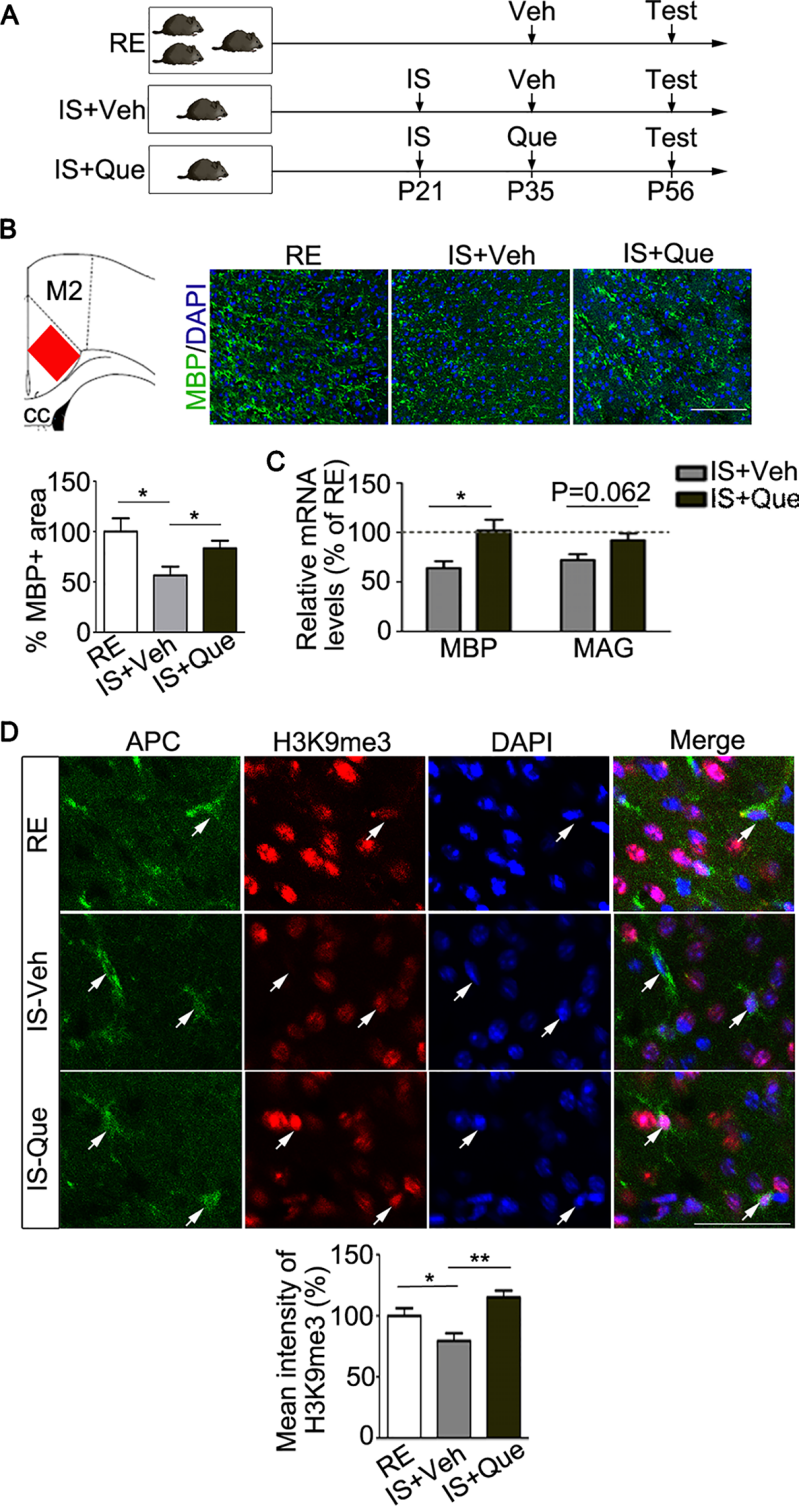
Analyses of myelin and histone methylation in oligodendroglial cells. **(A)** Experimental scheme. Mice were group-housed for 8 weeks or isolated for 5 weeks (from P21 to P56) followed by daily treatment of vehicle (ddH2O) or quetiapine for 3 weeks (from P35 to P56). At P56, mice were tested for behavior and sacrificed for morphological analyses. RE, group-housed in a regular environment; IS, social isolation; Veh, vehicle; Que, quetiapine. **(B)** Left panel, a scheme that indicated the cortex region where histological analysis for myelination was performed (red rectangle). Right panel, representative images of immunostaining of MBP showing myelinated fibers in prefrontal cortex (PFC) of mice. Bar graph shows the percentage of the MBP positive immunostaining area in the PFC (n = 6 for each group). Scale bars = 100 µm. **(C)** Quantification of transcripts of MBP and MAG in PFC, which is normalized to RE controls (dashed line) (n = 4 for each group). **(D)** Confocal images of H3K9me3 (red) and APC (green) in oligodendroglias in PFC of group-housed mice (RE), isolated mice treated with vehicle (IS-Veh) or quetiapine (IS-Que). Scale bar = 40µm. Bar graph shows quantification of H3K9me3 immunoreactivity in APC positive oligodendroglias in the PFC (n = 34 cells from 5 mice in RE group, n = 52 cells from 6 mice in IS-Veh group, n = 52 cells from 6 mice in IS-Que group). Data are expressed as mean ± SEM. *, p < 0.05; ** P < 0.01.

To test the hypothesis that quetiapine could reverse the myelin deficits result from social isolation, the socially isolated mice were orally treated with vehicle (ddH2O) or quetiapine (**Figure 2A**). As we know, PFC was a brain region functionally relevant to the control of social behavior (27). Recent study found that PFC was a specific region that displayed myelin deficiency in socially isolated mice as compared with other brain regions (i.e., nucleus accumbens, corpus callosum, and cerebellum) (7). Therefore, we focused on the analysis of myelination in the PFC in the present study. As shown in **Figure 2B**, immunofluorescence analysis of MBP-stained myelinated fibers was performed as previously reported (16). Strikingly, the quantification analysis revealed that the positive area of fluorescence labeling of MBP was significantly reduced in the PFC of the vehicle-treated isolated mice, as compared with group-housed mice in a regular environment (P = 0.021), while the positive area of fluorescence labeling of MBP in isolated mice treated with quetiapine was significantly higher than vehicle-treated group (P = 0.043) (**Figure 2B**). Moreover, we detected a significant increase of MBP transcript (P = 0.024) and a trend toward increased MAG transcript (P = 0.062) in PFC after quetiapine treatment (**Figure 2C**). These results demonstrated that quetiapine could rescue social isolation-induced myelin defect in the PFC.

### Quetiapine Increases Histone Methylation in Oligodendroglia of Socially Isolated Mice

Our previous studies have demonstrated that quetiapine could also promote OL maturation, which is a key process before myelin sheath formation (17–19). As reported, the level of repressive histone methylation, a marker of heterochromatin state, is necessary for the progression of the OL differentiation (28). In this study, we further examined the changes of a repressive histone methylation (H3K9me3) in OLs from socially isolated mice after quetiapine treatment. Consistent with previous study (16), we found that social isolation caused a decrease of H3K9me3 in mature OLs in the PFC. Interestingly, quetiapine significantly reversed the decrease of H3K9me3 immunoreactivity in mature OLs (**Figure 2D**). In fact, quetiapine treatment also significantly enhanced the level of H3K9me3 in mature OLs located in corpus callosum (data not shown). These results strongly suggest that quetiapine could favor chromatin compaction through regulating histone methylation status in OLs.

### Quetiapine Rescues Social Avoidance Behavior in Socially Isolated Mice

**Figure 3:**
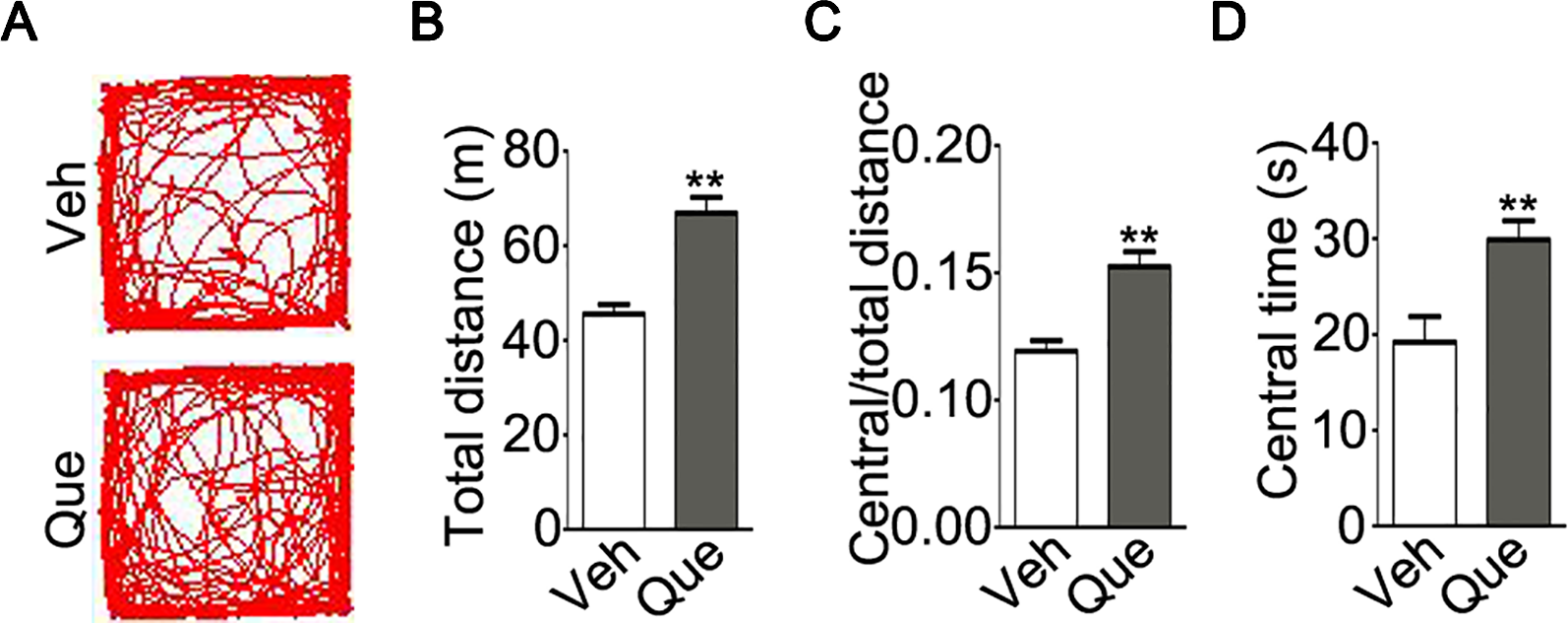
Analyses of locomotor activity in socially isolated mice after quetiapine treatment. **(A)** An example of the travel pathway of tested mice in open field test. Graphs show total distance **(B)**, ratio of central to total distance **(C)** and center time **(D)** in isolated mice treated with vehicle (Veh, n = 13) or quetiapine (Que, n = 13). Data are expressed as mean ± SEM. **P < 0.01.

We then tested the effect of quetiapine on above-mentioned maladaptive behaviors. After quetiapine treatment, we firstly analyzed mice locomotive activity in the open field test as shown in **Figure 3A**. Surprisingly, the isolated mice treated with quetiapine traveled a longer total distance as compared with isolated mice treated with vehicle (**Figure 3B**, P < 0.001). Moreover, the isolated mice treated with quetiapine showed higher ratio of traveled distance within central area to total traveled distance (**Figure 3C**, P < 0.001) and spent a longer time in the central area (**Figure 3D**, P = 0.004). These results indicate that quetiapine could increase locomotor activity of the socially isolated mice.

**Figure 4:**
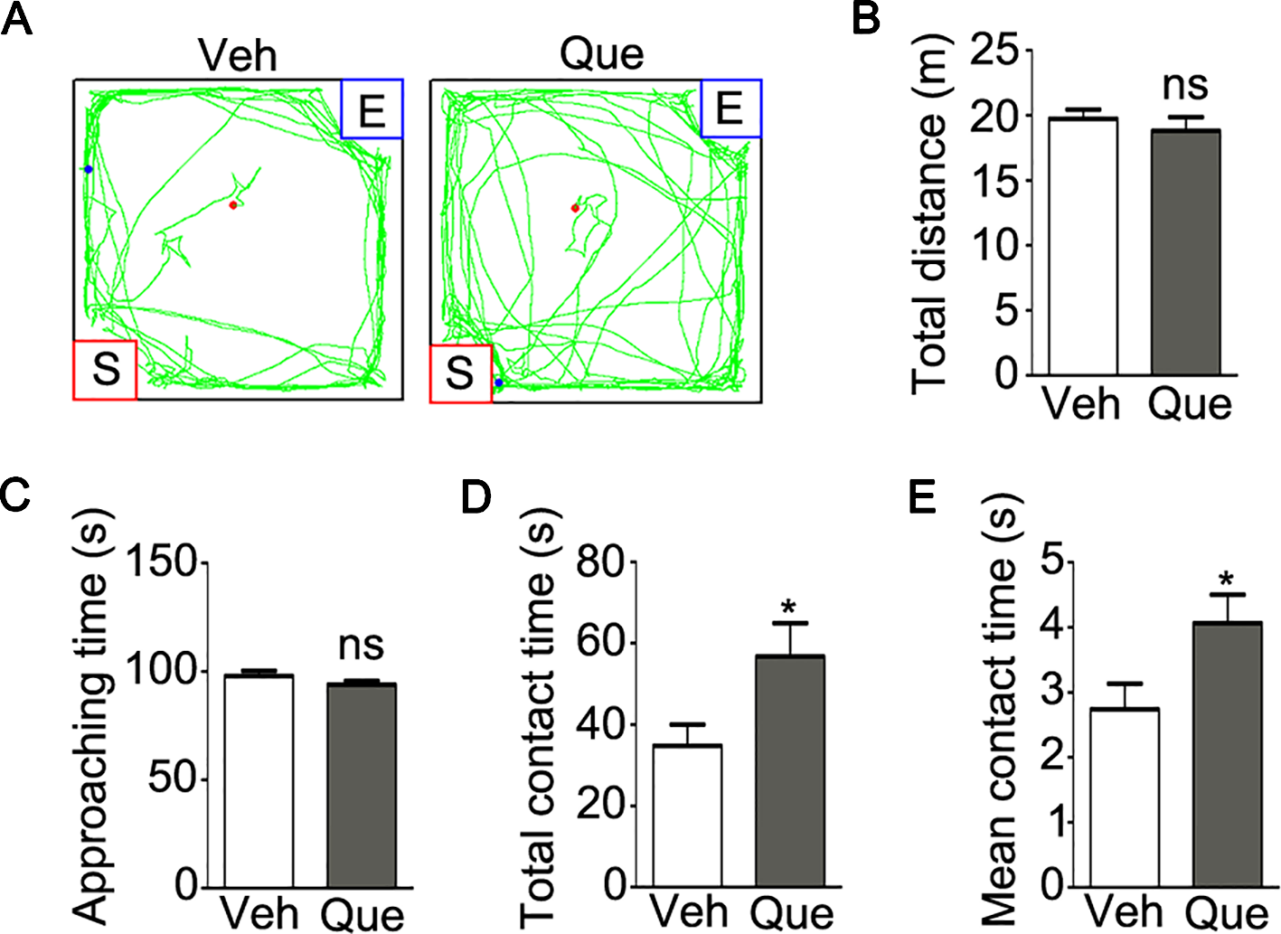
Analyses of sociability in isolated mice after quetiapine treatment. **(A)** an example of the travel pathway of tested mice in social interaction test. Graphs show the total distance traveled **(B)**, duration of approaching to the stranger mice **(C)**, duration of total contact **(D)** and mean duration for each contact **(E)** in isolated mice treated with vehicle (Veh, n = 12) or quetiapine (Que, n = 13). Data are expressed as mean ± SEM. *P < 0.05; ns, no significant difference.

Moreover, as shown in **Figure 4A**, we analyzed the mice social interaction behavior after quetiapine treatment. Firstly, no significant difference was found in the total traveled distance (**Figure 4B**, P = 0.475) or the duration of approaching the stranger mice (**Figure 4C**, P = 0.174) between the isolated mice treated with quetiapine and the isolated mice treated with vehicle. However, the quetapine treatment significantly increased the total duration of contacting with the stranger mice (**Figure 4D**, P = 0.037), as well as the mean duration for each contact (**Figure 4E**, P = 0.036). These results indicate that quetiapine was sufficient to rescue the social withdrawal behaviors under mild stress of social isolation.

## Discussion

In this study, we employed a mouse model through social isolation, which can led to myelin deficits in the PFC and impair the sociability of mice. We found that quetiapine can modulate the status of histone methylation in OLs, and rescue myelin deficiency in the PFC of the adult mice subjected to social isolation. Importantly, quetiapine treatment was sufficient to rescue social avoidance behavior in socially isolated mice.

Early social isolation in neonatal or juvenile animals result in behavioral abnormalities (6, 7, 29), but it is unclear whether this form of plasticity persists during adolescence, which is a peak time for the onset of psychiatric disorder, represented by schizophrenia (24). In this study, social isolation during a novel adolescent period rang from P21 to P56 led to a social withdraw behavior, and a trend of locomotive abnormalities in mice (**Figure 1**), that may provide us an ideal mouse model to dissect the effect of social isolation on sociability, one of the key negative symptoms of schizophrenic. In fact, it is widely accepted that exposure to stress altered neuronal activity in the PFC and resulted in psychiatric disorder-like behaviors in rodents (30–32).

Consistent with previous report that the lack of social experience in the postnatal early development impaired OL maturation and caused myelin deficits in the PFC and behavioral alterations in juvenile and adult mice (6, 7), we found that social isolation during adolescence also impaired myelination in the PFC (**Figure 2**). To be noted, neuronal activity has been widely recognized to affect OL development and function (9). Therefore, a potential explanation for the myelin deficits in the PFC from socially isolated mice could be decreased neuronal activity in this brain region.

In this study, quetiapine successfully rescue the myelin deficits in the PFC caused by social isolation (**Figure 2**), which is consistent with previous evidence that quetiapine could promote OL genesis and remyelination (17–19). Although the underlying mechanism is uncertain, we demonstrated that quetiapine was capable of enhancing the level of H3K9me3 in OLs (**Figure 2**). This is the first time to show a possibility that quetiapine could alter chromatin compaction, an important regulation mechanism for OL differentiation and myelination (33). However, whether and how quetiapine could interfere with other epigenetic programs, such as histone acetylation, chromatin remodeling, needs more investigation.

Moreover, a trend of lower locomotive activity was found in adolescent socially isolated mice, even though the difference of total distance and center time did not reach significance. Quetiapine treatment, however, increased locomotive activity (**Figure 3**), which is in agree with previous study report (34). In fact, antipsychotics usually have sedative effects which may affect the movement of mice (35). To be noted, the increased locomotive activity may result from functional alterations of motor neuron and/or GABAergic interneurons after quetiapine treatment (36, 37). More strikingly, quetiapine was sufficient to rescue the social avoidance behavior in socially isolated mice (**Figure 4**), thereby potentially overcoming the lack of neuronal activity in the PFC and consequent behavioral alterations. In addition, the effect of quetiapine on behavioral consequence could result from other brain regions except for the PFC, even though PFC is a brain region that is functionally relevant to the control of social behavior (27). To fully understand the effect of quetiapine on the brain function of socially isolated mice, more behavioral tests, such as the motor skill and cognitive function detection may be needed in the future study.

In summary, we found that quetiapine could modulate histone methylation in OLs and rescue myelin deficits and social withdraw of adult mice caused by mild stress of social isolation. Those results provide a new insight into the pharmacological mechanism of atypical antipsychotic quetiapine and confirm the idea that modulating epigenetic status in OLs could be a novel therapeutic target for white matter etiology of mental illnesses.

## Data Availability Statement

All datasets generated for this study are included in the article.

## Ethics Statement

All animal experiments were performed according to an approved protocol from Third Military Medical University Institutional Animal Care and Use Committee.

## Author Contributions

XC and LX designed the study. XC and HL acquired and analyzed the data. JG, XW, and GY also acquired the data. TL, XL and BY also analyzed the data. XC and LX wrote the article, which all other authors reviewed. All authors approved the final version for publication.

## Funding

This work is supported by the National Natural Science Foundation of China to LX (NSFC 31671117, 31921003).

## Conflict of Interest

The authors declare that the research was conducted in the absence of any commercial or financial relationships that could be construed as a potential conflict of interest.
